# Etiology, Risk Factors and Clinical Outcomes in Infective Endocarditis Patients Requiring Cardiac Surgery

**DOI:** 10.3390/jcm11071957

**Published:** 2022-04-01

**Authors:** Kristians Meidrops, Franziska Johanna Burkhardt, Janis Davis Osipovs, Eva Petrosina, Valerija Groma, Peteris Stradins

**Affiliations:** 1 Riga Stradins University, LV-1007 Riga, Latvia; franziskajohannab@gmail.com (F.J.B.); janis.davis.osipovs@gmail.com (J.D.O.); eva.petrosina@rsu.lv (E.P.); gromavalerija@gmail.com (V.G.); peteris.stradins@stradini.lv (P.S.); 2 Centre of Cardiac Surgery, Pauls Stradins Clinical University Hospital, LV-1002 Riga, Latvia

**Keywords:** infective endocarditis, outcome, echocardiography, cardiac surgery

## Abstract

Background: Infective endocarditis, which may be caused by various microbial agents, severely affects the innermost layer of the heart and often leads to poor clinical outcomes. The purpose of this study was to investigate the etiology, risk factors and short and long-term outcomes of infective endocarditis caused by various bacterial agents in patients requiring cardiac surgery. Methods: One hundred and forty-four patients aged 18 years or above with indications for cardiac surgery due to *S. aureus*, *Streptococcus* spp., *E. faecalis* or coagulase-negative staphylococci caused infective endocarditis were included in this study. Results: *S. aureus*, *Streptococcus* spp., *E. faecalis* and coagulase-negative staphylococci were the causative agents of infective endocarditis in 44 (30.6%), 35 (24.3%), 33 (22.9%) and 32 (22.2%) patients, respectively. The presence of bicuspid aortic valve was the most common predisposing factor confirmed in 19 (23.5%), whereas intravenous drug usage was the most common in 17 (11.8%) patients. No significant differences in intrahospital mortality due to infective endocarditis caused by various bacterial agents were found, however, the worsening of long-term prognosis of endocarditis caused by *S. aureus* when compared to *E. faecalis* was confirmed (*p* = 0.03). The presence of *S. aureus* was associated with significantly higher rates of embolic complications (*p* = 0.003). The presence of coagulase-negative staphylococci was associated with prosthetic valve endocarditis (*p* = 0.015) and perivalvular complications (*p* = 0.024). Conclusions: In contrast to *E. faecalis*, the presence of *S. aureus* determines the worsening of the long-term mortality from infective endocarditis. Perivalvular complications are associated with the presence of coagulase-negative staphylococci.

## 1. Introduction

Infective endocarditis (IE) is a life-threatening infection of the endocardium with high morbidity and mortality rates despite improvement in diagnostics and treatment [1]. The most commonly affected valves in descending order are the mitral, aortic, the combination of mitral and aortic valves, tricuspid and the pulmonic valve. Sometimes infection spread is mixed and affects the right and left side of the heart [2]. The annual incidence of IE is up to 10/100,000 in the general population and carries a mortality of up to 30% at 30 days [3]. IE is mainly caused by bacteria, however, occasionally, fungi are also the cause of the disease. The most common IE causative bacteria are *S. aureus*, *Streptococcus* spp., *Enterococcus* spp. and coagulase-negative staphylococci (CoNS) [4]. 

The diagnosis of IE is based on the modified Duke criteria, where the detection of causative microorganisms and echocardiographic imaging play a central role [5]. The identification of a bacterial agent is essential for both diagnostics of IE and effective targeting of a pathogen-specific antibacterial treatment. Recent studies suggest a delay in diagnosis can be associated with poorer outcomes [6].

Echocardiography is the recommended imaging method to visualize the manifestation of the IE process. Commonly, it is used for the detection of vegetation formation, valvular dysfunction, septal defects, fistula formation or other defects of cardiac structures [7]. Transthoracic (TTE) and transoesophageal (TOE) echocardiographic imaging techniques are used for the detection of endocardial involvement in IE. The highly sensitive and specific TOE provides better imaging quality and commonly follows TTE examination when a diagnosis of IE is suspected. Furthermore, TOE is performed in cases of prosthetic valve IE and poor quality TTE, to rule out local complications of infection [8].

The latest American College of Cardiology/American Heart Association (ACC/AHA) guidelines on the management of patients with valvular heart disease state that the fast evaluation conducted by a multidisciplinary team, followed by either referral to or consultation with a Primary or Comprehensive Valve Center plays a crucial role in the successful treatment of patients with severe valvular heart disease [9]. Surgery is required in up to 50% of all IE patients. The indications for surgical treatment of IE are specified in the international guidelines and address questions essential to cardiac surgeons before, during and after the operation [8,10]. Mostly, cardiac surgery is required in case of heart failure, severe valvular dysfunction, prosthetic valve infection, recurrent embolization, large-sized and mobile vegetations and persistent sepsis despite adequate antibiotic therapy for more than 5–7 days [10]. Technically, in IE presented with complex valvular damage and spread of infection, cardiac surgery faces many challenges which are associated with high risk, especially in severely ill patients with multiorgan failure [11]. The presence of *S. aureus* is associated with higher early mortality risk and severe complications such as congestive heart failure and embolic events; moreover, mortality appears to correlate with the presence of *S. aureus* [12,13]. The postoperative prognosis of these patients is often obscure. Over time, several prognostic models for postoperative mortality after cardiac surgery in IE patients were developed [14]. Recently, other researchers have explored these prognostic models and confirmed that only a few prognostic models have not displayed a high risk of bias. In these predictive models for mortality, the case of bias is likely to be associated with small sample sizes and poor statistical analysis of available data. Apparently, the determination of prognosis in surgically treated IE patients remains to be a challenge. Furthermore, other authors suggest worse clinical outcomes in patients with IE caused by *Staphylococcus* spp. [14]. 

Although some evidence suggests the presence of factors affecting the outcomes in surgically treated IE patients, the association between clinical outcomes and echocardiographic manifestations of IE caused by various microorganisms remains insufficiently studied [15,16,17]. Moreover, in the current era of cardiac surgery, the etiology and outcomes may well be changing over time and geographic location [12,18].

## 2. Materials and Methods

Medical records of 253 patients clinically presented with IE caused by various bacterial agents and treated surgically at Pauls Stradins Clinical University Hospital, Riga, Latvia, between January 2016 and September 2020 were used for further selection and assessment in this retrospective study. Long-term mortality was assessed using the Latvian population register. The study was approved by the Ethical Committee of Pauls Stradins Clinical University Hospital (Decision No. 230419-17 L) and conducted according to the Declaration of Helsinki. The necessity for informed consent was waived due to retrospective patients’ data analysis and the absence of any sensitive information.

### 2.1. Objectives of the Study and Inclusion Criteria

The aim of this study was to explore etiology, risk factors and clinical outcomes in IE patients undergoing cardiac surgery.

Patients were eligible for the study if they were aged 18 years or older and underwent cardiac surgery due to *S. aureus, Streptococcus* spp., *E. faecalis* and coagulase-negative staphylococci caused IE. Patients with an unknown pathogen or IE caused by other pathogens were excluded from the study due to the high heterogeneity of microbial agents. The analyzed data included information on patients’ characteristics, clinical outcomes, complications of IE, findings of echocardiographic imaging, and blood culture tests for microbial diagnostics.

A diagnosis of IE based on modified Duke criteria was jointly established by cardiologists, cardiac surgeons, and other specialists. All patients included in this study are classified as definite IE by the use of the modified Duke criteria [5]. Since Pauls Stradins Clinical University Hospital is the only institution providing adult cardiac surgery services in the country, a diagnosis of IE was made either on-site or at another hospital in Latvia, if a patient had been transferred for further treatment. Indications for cardiac surgery were based on 2015 ESC European guidelines for the management of infective endocarditis recommendations. Patients with a serious stroke were operated on only if the neurological prognosis was not judged as too poor. Patients with intracranial haemorrhage were not undergoing cardiac surgery on an urgent basis but underwent conservative treatment with surgery postponed for ≥1 month.

### 2.2. Diagnostic Techniques Used for the Detection of Endocardial Involvement and Embolism

Both TTE and TOE were applied as suggested in the 2015 ESC European guidelines for the management of infective endocarditis as the methods of choice for the diagnosis of IE. Preoperatively, a major part of the IE patients were examined using both echocardiographic imaging techniques, TTE and TOE, whereas intraoperatively, all IE patients undergoing cardiac surgery were imaged using TOE. In case of any discrepancies of imaging data obtained by the use of TTE and TOE, TOE indices were used and submitted to further statistical analyses in this study.

Embolic events were diagnosed with the use of computed tomography (CT) or magnetic resonance imaging, based on clinical necessity.

### 2.3. Methods of Microbiological Investigation

The IE patients’ blood and valve tissue specimens obtained at the time of surgery were further submitted to conventional microbiological analyses used to detect a pathogen. No serological tests or other extended diagnostic strategies were used for the detection of IE causative microorganisms. In the present study, antibiotic therapies were regimented to the recommendations of the 2015 ESC European guidelines for the management of infective endocarditis and were based on the causative microorganism confirmed and antibacterial susceptibility.

### 2.4. Statistical Data Analysis

The statistical analyses were performed using the RStudio Version 1.4.1717. Descriptive statistics median and interquartile range were used for quantitative variables, whereas count and relative count were used for categorical ones. Inferential statistical methods included the Kruskal–Wallis rank-sum test, Pearson’s Chi-squared test and Fisher’s Exact Test for Count Data with simulated *p*-value (based on 2000 replicates) for group comparisons accordingly to their assumptions. The Kaplan–Meier method was used for visualization of survival curves, whereas the log-rank test and Peto and Peto modification of the Gehan–Wilcoxon test was used for group comparison. A *p*-value of less than 0.05 was considered statistically significant.

## 3. Results

### 3.1. General Information

During the study timespan, 253 patients were diagnosed with IE, which led to cardiac surgery. The rate of blood culture-negative IE was 35.17%. The most common causative microorganisms were *S. aureus, E. faecalis, Streptococcus* spp. or CoNS, confirmed in 144 blood culture-positive patients. Other pathogens were present in 20 patients only (7.91%). Among all IE patients, 70 (48.61%) patients were transferred from other hospitals. All IE patients fulfilled two major Duke criteria. There were 131 left- and 11 right-sided IE patients. There was a significant association found between the presence of right-sided IE and history of intravenous drug usage (*p* < 0.001). Two subjects presented both left- and right-sided IE. Patients’ characteristics are displayed in Table 1. In the present study, the most common indication for cardiac surgery on IE patients was heart failure due to valvular dysfunction combined with the prevention of embolism. Other indications are summarized in Table 2. The indications for cardiac surgery combined with a surgery type and surgical times depending on the IE causative agent are summarized in Table 3. Overall, there were 115 (79.9%) and 29 (20.1%) native and prosthetic valve endocarditis cases, respectively. Infections were mostly community-acquired. Only 10 (6.9%) of all IE cases were classified as hospital acquired. Among 44 *S. aureus* caused IE patients, only three subjects were methicillin-resistant. Most of the *Streptococcus* spp. were classified as the *Viridans* group streptococci and were confirmed in 23 (65.7%) patients. The Beta-haemolytic group streptococci infection was present in 10 (28.6%) patients, of whom six were group A and four group B streptococci. Two patients (5.7%) presented with *S. pneumoniae* caused IE.

Embolic complications were diagnosed in IE caused by all studied pathogens. When specified, embolic complications of IE included the central nervous system (CNS), spleen and kidney in 13.2, 11.1 and 6.3% of IE patients, respectively. IE subjects with embolic events presented larger vegetations; however, the difference was not statistically significant (17 vs. 15 mm, *p* = 0.219).

### 3.2. Clinical Outcomes

**Figure 1:**
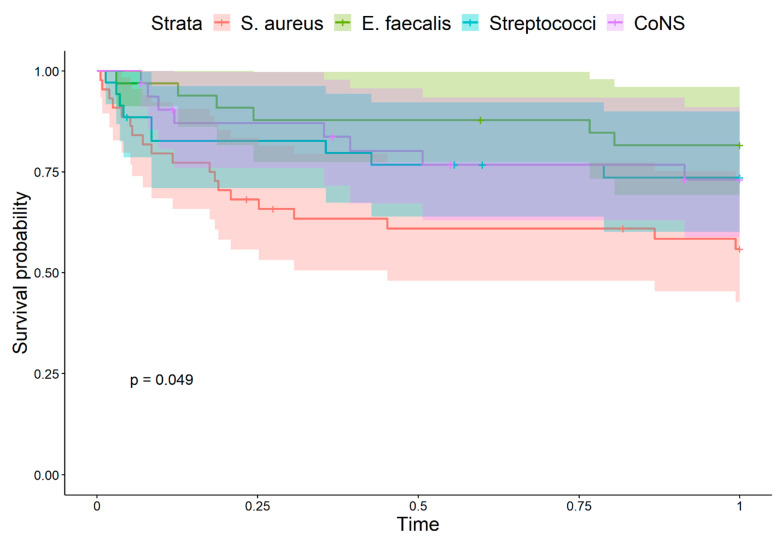
Kaplan–Meier 1-year survival curves when comparing IE groups presented with different causative microorganisms. *p*-Value represents the overall differences.

**Figure 2:**
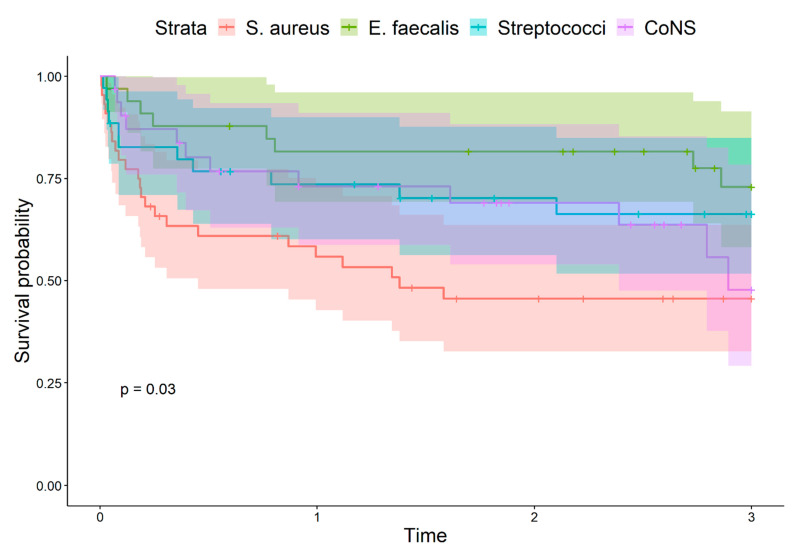
Kaplan–Meier 3-year survival curves when comparing IE groups presented with different causative microorganisms. *p*-value represents the overall differences.

Despite no statistical difference found in intrahospital mortality, in the calculated length of intrahospital and ICU stay, significant differences appeared after one and three years, with the highest mortality of *S. aureus* and lowest mortality of *E. faecalis* caused IE (Figure 1 and Figure 2). Commonly, embolic complications were observed in the group of IE caused by *S. aureus.* Furthermore, the presence of *S. aureus* infection was associated with embolism to the CNS (*p* = 0.005), confirmed in 25% of IE patients (Table 3). Patients with IE caused by *S. aureus* had diabetes more often when compared to subjects with IE caused by other microbial agents (16.3 vs. 9.1%, *p* = 0.251), however, the difference didn’t reach statistical significance.

The group of IE caused by *E. faecalis* constituted of the oldest subjects when compared to the other groups, with a median age of 65.0 years. The 1-year survival and 3-year survival was the highest, in contrast to *S. aureus* where survival was the lowest.

Patients presented in the *Streptococcus* spp. group were the youngest—with a median age of 50.0 years. Furthermore, they commonly presented with native valve endocarditis. The presence of *Streptococcus* spp. was associated with more severe aortic regurgitation, and fistulas developed between the cardiac chambers (Table 4).

In the CoNS group, the highest incidence of prosthetic valve endocarditis and perivalvular complications were observed. Patients with CoNS caused IE presented with higher BMI when compared to patients with endocarditis caused by other bacterial agents (26.8 vs. 24.6 kg/m^2^, *p* = 0.003), however, no significant association with type 2 diabetes was found (9.8 vs. 13.3%, *p* = 0.522). Echocardiographic parameters did not differ significantly, except for perivalvular complications, when microbial agents were compared (Table 5).

### 3.3. Comorbidities, IE Predisposing and Risk Factors

The most common comorbidities confirmed in IE patients were chronic kidney disease, co-existing oncology, chronic obstructive pulmonary disease, diabetes mellitus, and chronic viral hepatitis C infection diagnosed in 43 (29.9%), 9 (6.3%), 7 (4.9%), 17 (11.8%) and 14 (9.7%) patients, respectively, with the latter being prevalent among intravenous drug users.

The presence of bicuspid aortic valve was the most common IE-predisposing factor recognized in the given study and confirmed in 19 (23.5%) patients, whereas intravenous drugs usage was confirmed in 17 (11.8%) patients. The presence of previously diagnosed IE and rheumatic valve disease were confirmed in 2 patients (1.4%).

When comparing patient characteristics of survivors and non-survivors, the non-survivors presented with significantly more pronounced worsening of left ventricle EF, more frequent embolic events, increased ICU length of stay, longer CPB, aortic cross-clamp, mechanical lung ventilation, catecholamine administration time, higher SOFA score and postrenal kidney failure, requiring dialysis (Table 6). In turn, when comparing characteristics of IE patients with severe vs. not severe valve regurgitation, we found that the size of vegetation was significantly larger in the group with severe valve regurgitation (*p* = 0.044). Similarly, when comparing outcomes in patients with perivalvular complications, the only statistically significant difference confirmed was related to the frequency of aortic regurgitation: IE patients with perivalvular complications had significantly more aortic regurgitation (*p* < 0.001).

## 4. Discussion

In this study, etiology, risk factors and clinical outcomes of IE caused by various bacterial agents in surgically treated patients were analyzed.

It is increasingly clear that the presence of *S. aureus* infection often correlates with worsening of clinical outcomes in IE. During the given five year timespan in our institution, *S. aureus* was the most frequently detected causative microorganism. These findings are supported also by other authors suggesting that *S. aureus* is the leading cause of IE in most parts of the world [1,19,20]. *S. aureus* is a pathogen known for its aggressive nature; often, it is associated with severe clinical presentation [21]. There are results from other authors reporting on subclinical cerebral embolism in cases of left-sided IE and confirmed in 30% of the patients [22]. It is worth mentioning that embolism to the CNS was most often observed in *S. aureus* caused IE. Simultaneously, there was no significant difference found in the vegetation size assessed for the *S. aureus* group and compared to the other bacteria groups.

In this study, except for echocardiography, other imaging was performed due to clinical necessity only. No systematic imaging for the detection of embolism was performed thus potentially leaving a gap in complete coverage of embolic complications. We found that there were significantly more embolic events in subjects with intrahospital death when comparing survivors to non-survivors, thus emphasizing the role of embolism prevention. The study suggests the size of vegetation assessed by using echocardiography also plays a pivotal role in predicting embolic events. Similarly, other authors reported that patients with vegetations larger than 10 mm are at high risk of embolism; furthermore, patients with vegetations larger than 25 mm in the setting of right-sided IE are predisposed to reoccurrence [23,24]. The importance of measurements related to the size of vegetations in the early prognostic assessment of IE aiming at a reduction of the development of embolic events is demonstrably crucial [25].

Despite the lack of difference in the intrahospital mortality when IE caused by different bacterial agents is compared, the study suggests the worse long-term clinical outcomes for endocarditis in the cases of *S. aureus.* This statement is in agreement with the results obtained by other authors suggesting the presence of *S. aureus* is associated with poor outcomes and high mortality in IE [26]. Opposingly, as demonstrated in this study, *E. faecalis* caused IE presents a less severe course, complications, and better outcomes in surgically treated patients.

When data on the specificity of valvular dysfunction detected by echocardiography are summarized, a significant association of severe aortic insufficiency and fistula formation between the cardiac chambers with the presence of *Streptococcus* spp. comes to light. Furthermore, to better understand the anatomical and dimensional peculiarities of a disease, IE patients presented with perivalvular abscess and fistula formation confirmed by the use of echocardiography underwent cardiac CT angiography. Among IE patients used in this study, three native valve subjects presented with fistulas connecting the aorta to the right atrium, and one subject with the pathological connection between the left ventricle to the aorta. Other fistula cases were associated with prosthetic valve endocarditis. During surgery, larger abscesses of fistulas were closed with the use of xenopericardium or other materials, depending on the situation and anatomy. No homografts were used. The presence of more severe pathology exemplified by fistula formation between cardiac chambers is likely to explain a relatively high intrahospital mortality observed in *Streptococcus* spp. group. Furthermore, the comparison of survivors and non-survivors confirmed the significantly shorter time of aortic cross-clamp and CPB in the formers, thus leading to the suggestion that complex surgery with longer operative time has a worse prognosis. However, the probability that the aforementioned results are likely to be associated with the sample bias due to a rather small number of patients, is also high. Complications, associated with *Streptococcus* spp. and demonstrated in our study, partly could be explained by a relatively high proportion of beta-haemolytic streptococci infection confirmed in 10 (28.6%) patients possibly having an impact on the course of the disease. A long time till diagnosis and treatment as well as a long time to referral center could also serve as an explanation of the worse results for streptococcal IE.

Interestingly, among echocardiographic findings and parameters analysed in the given study, none appeared to be specifically linked to a causative bacterial agent. Overall, the native valves were more often affected (79.9%) compared to the prosthetic valves (20.1%). Furthermore, CoNS were the most frequent cause of prosthetic valve IE, and these findings are in line with the data published by other authors suggesting that CoNS accounts for approximately 60% of all cases [27]. The aforementioned results have a close association with the statistically more frequent observation of perivalvular complications of IE with the CoNS as a causative agent in the present study. These findings are also supported by other authors [16,17].

The duration of mechanical lung ventilation and catecholamine administration or necessity for postoperative dialysis and higher SOFA scores were found to be significantly higher among IE non-survivors, along with the longer CPB and cross-clamp times.

The findings of this study have to be considered in light of some limitations. The authors were focused on cardiac surgery patients only. Conservatively treated patients were not included in the present study. Most of the patients analysed in the given study presented with active infection, however, they were operated on at the different settings of urgency. Moreover, the type of operation, not specified and analyzed in the present study, could have an impact on the results obtained. Most of the operations performed were valvular replacement and debridement of the infectious process. More complex repair techniques were applied to the minority of IE patients in cases of severe infection. The relatively small sample size is another limitation of the given study. Possibly, new data characteristics of clinical outcomes could come to light when specifying heterogenous streptococcal species and their caused IE [28]. Other authors have explored the clinical course and prognosis of IE caused by different streptococcal species and failed to show significant discrepancies [29]. The cause of death, when related to long-term mortality, was not specified in the present study. Finally, the authors did not explore the impact of additional medical conditions, comorbidities and therapies of the IE patients, which might bring some new data and interpretations.

## 5. Conclusions

In contrast to *E. faecalis*, the presence of *S. aureus* determines the worsening of long-term mortality from infective endocarditis. Perivalvular complications are associated with the presence of coagulase-negative staphylococci.

## Figures and Tables

**Table 1:** Patients’ characteristics.

Characteristics	*S. aureus*, N = 44	*Streptococcus* spp., N = 35	*E. faecalis*, N = 33	CoNS, N = 32	*p*-Value
Age, years	58.5 (45.0–66.0)	50.0 (37.0–65.5)	65.0 (56.0–73.0)	56.0 (48.2–69.2)	**0.037**
Males, %	77.3	85.7	87.9	68.8	0.195
BMI, mean, (kg/m^2^)	24.7 (21.1–27.1)	24.7 (22.1–27.6)	24.4 (23.5–29.3)	26.6 (24.7–30.9)	**0.031**
EuroScore II risk, %	4.7 (2.2–7.0)	2.9 (2.0–4.9)	3.1 (2.0–8.0)	4.4 (2.1–10.9)	0.237
Native valve IE, %	82.5	94.1	78.1	62.5	**0.015**
Left-sided IE, %	79.5	97.1	96.9	90.6	0.059
Right-sided IE, %	182	2.9	3.1	6.3	0.136

Table note: CoNS—coagulase-negative staphylococci; BMI—body mass index; IE—infective endocarditis. *p*-Values <0.05 highlighted in bold.

**Table 2:** Patients’ indications for cardiac surgery due to IE.

Indication	N (%)
Heart failure due to valvular dysfunction	19 (13.19%)
Prevention of embolism	6 (4.17%)
Uncontrolled infection	3 (2.08%)
Heart failure due to valvular dysfunction and prevention of embolism	67 (46.53%)
Heart failure due to valvular dysfunction and uncontrolled infection	21 (14.58%)
Prevention of embolism and uncontrolled infection	4 (2.78%)
Heart failure due to valvular dysfunction, prevention of embolism, and uncontrolled infection	24 (16.67%)

Table note: Indications for cardiac surgery according to 2015 ESC European guidelines for the management of infective endocarditis.

**Table 3:** Type of surgery, surgical times and indications for surgery stratified according to IE causative agent.

Characteristic	*S. aureus*	*Streptococcus* spp.	*E. faecalis*	CoNS	*p*-Value
Type of surgery:					
Valvular plasty	5 (11.6%)	2 (6.1%)	2 (5.7%)	2 (6.5%)	0.819
Biological valve implantation	34 (77.3%)	29 (87.9%)	27 (77.1%)	29 (93.5%)	0.178
Mechanical valve implantation	6 (13.6%)	3 (9.1%)	8 (22.9%)	3 (9.7%)	0.372
CPB time, minutes	101 (74, 135)	88 (75, 139)	94 (70, 138)	114 (93, 135)	0.569
Cross-clamp time, minutes	73 (50, 97)	62 (52, 94)	69 (51, 111)	80 (68, 104)	0.279
Indication for surgery:					
Heart failure	39 (88.6%)	32 (97.0%)	32 (91.4%)	27 (87.1%)	0.508
Embolism prevention	30 (68.2%)	22 (66.7%)	20 (57.1%)	28 (90.3%)	0.028
Uncontrolled infection	15 (34.1%)	13 (39.4%)	12 (34.3%)	11 (35.5%)	0.964

Table note: CoNS—coagulase-negative staphylococci; CPB—cardiopulmonary bypass.

**Table 4:** Clinical outcomes and complications of IE caused by various bacterial agents.

Characteristic	*S. aureus*	*Streptococcus* spp.	*E. faecalis*	CoNS	*p*-Value
Intrahospital stay, days	28.0 (19.0–42.0)	23.0 (15.5–31.0)	26.0 (17.0–37.0)	27.5 (22.0–35.0)	0.310
ICU stay, days	3.0 (1.0–9.8)	2.0 (1.0–5.0)	2.0 (1.0–4.0)	2.0 (1.0–4.0)	0.230
Embolic complications, %	42.5	8.8	34.4	15.6	**0.003**
CNS embolism, %	25.0	0.0	18.8	9.4	**0.005**
Spleen embolism, %	12.5	8.8	15.6	9.4	0.809
Kidney embolism, %	7.5	0.0	12.5	6.2	0.203
Intrahospital mortality, %	22.7	17.6	9.4	9.4	0.324
Fistula between cardiac chambers, %	2.7	15.2	0.0	3.2	**0.049**
Severe (grade 3–4) aortic regurgitation, %	20.5	57.1	27.3	28.1	**0.004**
Severe (grade 3–4) mitral regurgitation, %	20.5	28.6	33.3	18.8	0.455
Severe (grade 3–4) tricuspid regurgitation, %	15.9	2.9	9.1	6.2	0.235
Predominantly aortic stenosis	6.8	8.6	12.1	21.9	0.249
Predominantly mitral stenosis	2.3	0.0	9.1	12.5	0.074

Table note: CoNS—coagulase-negative staphylococci; ICU—intensive care unit; CNS—central nervous system. *p*-Values <0.05 highlighted in bold.

**Table 5:** Echocardiographic findings and parameters observed in IE caused by various microorganisms.

Characteristic	*S. aureus*	*Streptococcus* spp.	*E. faecalis*	CoNS	*p*-Value
TOE performed preoperatively, %	68.4	75.0	79.3	85.7	0.414
Size of vegetation, mm	16.0 (12.5–20.0)	14.0 (10.0–17.0)	15.0 (13.0–22.5)	15.0 (10.0–20.0)	0.250
Perivalvular complications, %	15.8	27.3	3.4	32.3	**0.024**
EF, %	59.0 (52.0–60.0)	56.5 (54.2–60.0)	58.0 (50.0–65.0)	60.0 (55.0–65.0)	0.541
EDD, mm	53.0 (50.0–58.0)	56.5 (52.0–59.8)	53.0 (50.0–59.5)	53.0 (48.0–58.0)	0.272
ESD, mm	35.0 (31.0–38.0)	37.0 (33.0–38.8)	34.5 (30.5–37.2)	35.0 (31.0–39.0)	0.275
IVS, mm	11.0 (9.5–13.0)	10.0 (9.0–12.0)	10.0 (9.0–11.5)	11.0 (9.0–14.0)	0.496
TAPSE, mm	22.0 (18.5–25.5)	21.0 (18.0–24.0)	21.0 (17.5–23.5)	20.0 (18.5–24.2)	0.748
LAVI, mL/m^2^	45.0 (31.0–59.0)	43.0 (37.0–51.0)	38.0 (31.8–55.2)	47.0 (36.2–57.0)	0.817
Ascending aortic diameter, mm	34.0 (32.2–35.8)	37.0 (35.0–44.0)	35.0 (33.2–37.5)	35.0 (31.0–37.0)	0.245
RVSP, mm/Hg	40.0 (35.0–50.0)	40.0 (33.5–55.0)	38.0 (30.0–50.0)	35.5 (30.0–51.2)	0.406

Table note: CoNS—coagulase-negative staphylococci; TOE—transoesophageal echocardiography; EF—ejection fraction of left ventricle, EDD—end-diastolic diameter, ESD—end-systolic diameter, IVS—interventricular septum, TAPSE—tricuspid annular plane systolic excursion, LAVI—left atrial volume index, RVSP—right ventricular systolic pressure. *p*-Values <0.05 highlighted in bold.

**Table 6:** Comparison of microbiology and other characteristics among survivors and non-survivors IE patients.

Characteristic, N (%)	Survivors, N = 120 ^1^	Non-Survivors, N = 22 ^1^	*p*-Value ^2^
Microbiology			0.324
*S. aureus*	34 (28.3%)	10 (45.5%)	
*E. faecalis*	29 (24.2%)	3 (13.6%)	
*Streptococcus* spp.	28 (23.3%)	6 (27.3%)	
CoNS	29 (24.2%)	3 (13.6%)	
Age	57.0 (44.8–66.2)	67.0 (52.8–72.0)	0.081
Intrahospital stay, days	28.0 (19.0–37.2)	24.0 (13.5–31.0)	0.104
ICU stay, days	2.0 (1.0–4.0)	5.0 (3.0–13.0)	**<0.001**
Males	96 (80.0%)	17 (77.3%)	0.776
BMI, kg/m^2^	24.8 (22.6–28.4)	26.5 (24.7–30.0)	0.341
EuroScoreII risk	3.5 (2.0, 7.2)	5.9 (2.9, 11.8)	0.093
I/v drug use			0.472
No	104 (86.7%)	21 (95.5%)	
Yes	16 (13.3%)	1 (4.5%)	
EF of the left ventricle, %	60.0 (55.0–63.0)	55.0 (46.2–60.0)	**0.025**
TAPSE, mm	21.0 (18.0–24.0)	20.0 (17.2–23.5)	0.458
Size of vegetation, mm	15.0 (12.0–20.0)	12.5 (10.0–17.0)	0.144
Embolism			**0.037**
No	91 (77.1%)	11 (55.0%)	
Yes	27 (22.9%)	9 (45.0%)	
Perivalvular complications			0.240
No	100 (83.3%)	16 (72.7%)	
Yes	20 (16.7%)	6 (27.3%)	
Severe AR			0.890
No	80 (66.7%)	15 (68.2%)	
Yes	40 (33.3%)	7 (31.8%)	
Severe MR			0.758
No	89 (74.2%)	17 (77.3%)	
Yes	31 (25.8%)	5 (22.7%)	
Severe TR			0.220
No	107 (89.2%)	22 (100.0%)	
Yes	13 (10.8%)	0 (0.0%)	
CPB time, minutes	94 (73, 129)	153 (122, 196)	**<0.001**
Cross-clamp time, minutes	69 (52, 92)	107 (79, 113)	**<0.001**
COPD	5 (4.2%)	2 (9.1%)	0.300
CKD	36 (30.5%)	7 (31.8%)	0.903
Diabetes	11 (9.2%)	5 (22.7%)	0.134
Oncology	8 (6.7%)	1 (4.5%)	>0.999
SOFA score at the admission at the ICU	5.00 (4.00, 6.00)	8.00 (6.25, 9.00)	**<0.001**
Duration of mechanical lung ventilation	7 (4, 14)	36 (16, 76)	**<0.001**
Duration of catecholamine administration	8 (0, 24)	50 (30, 90)	**<0.001**
Postoperative dialysis	7 (5.9%)	6 (27.3%)	**0.006**

Table note: ^1^ n (%); Median (IQR); ^2^ Fisher’s exact test; Wilcoxon rank-sum test; Pearson’s Chi-squared test; CoNS—coagulase-negative staphylococci; ICU—intensive care unit; BMI—body mass index; i/v—intravenous; EF—ejection fraction; TAPSE—tricuspid annular plane systolic excursion; AR—aortic regurgitation; MR—mitral regurgitation; TR—tricuspid regurgitation; COPD—chronic obstructive pulmonary disease; CKD—chronic kidney disease; SOFA—Sequential Organ Failure Assessment. *p*-Values <0.05 highlighted in bold.

## Data Availability

Data are available from the corresponding author upon reasonable request, preferentially by e-mail: kristians.meidrops@stradini.lv.
